# Baricitinib in moderate to severe rheumatoid arthritis: dose-dependent efficacy and safety in a randomized clinical trial in a developing country

**DOI:** 10.1186/s41927-026-00638-8

**Published:** 2026-03-30

**Authors:** Md. Sadikul Islam, Minhaj Rahim Choudhury, Mohammad Abul Kalam Azad, Md. Abu Shahin, Syed Atiqul Haq, S. M. Zobair Hossain, Nabil Amin Khan, Md. Atiqur Rahman

**Affiliations:** 1 Department of Rheumatology, Rangpur Medical College Hospital, Rangpur, Bangladesh; 2 Department of Rheumatology, Bangladesh Medical University, Shahbagh, Dhaka, Bangladesh; 3 Rajshahi Medical College Hospital, Rajshahi, Bangladesh; 4 Department of Rheumatology, Dhaka Medical College Hospital, Dhaka, Bangladesh; 5 Central Police Hospital, Dhaka, Bangladesh

**Keywords:** Rheumatoid arthritis, Baricitinib, Methotrexate, Disease-modifying antirheumatic drugs, Randomized controlled trial

## Abstract

**Background:**

Patients with rheumatoid arthritis (RA) who have an inadequate response or intolerance to methotrexate require effective and safe alternative therapies. Baricitinib, a Janus kinase inhibitor, has shown efficacy in RA, but dose-dependent outcomes in developing countries remain limited.

**Objectives:**

To compare the efficacy and safety of baricitinib 2 mg versus 4 mg, both in combination with methotrexate, in patients with moderate-to-severe RA with an inadequate response to methotrexate.

**Methods:**

This was a 24-week, open-label, randomized controlled trial conducted in a developing country. Adult patients with moderate-to-severe RA and inadequate response or intolerance to methotrexate were randomly assigned (1:1) to receive either baricitinib 2 mg daily plus methotrexate 10 mg weekly or baricitinib 4 mg daily plus methotrexate 10 mg weekly. Primary outcome: proportion of patients achieving low disease activity (LDA) according to DAS28-CRP at week 24. Secondary endpoints included LDA by DAS28-ESR and Clinical Disease Activity Index (CDAI). The Simplified Disease Activity Index (SDAI), functional improvement assessed by the Bangla Health Assessment Questionnaire–Disability Index (B-HAQ), and changes in core set outcomes and acute-phase reactants. Safety outcomes were assessed throughout the study. Analyses were conducted on an intention-to-treat basis.

**Results:**

A total of 94 patients were randomized, with 47 per treatment group. At week 24, LDA by DAS28-CRP was achieved by 38 patients (84.4%) in the baricitinib 4 mg group compared with 18 patients (45.0%) in the baricitinib 2 mg group (*P* = 0.01). Remission alone occurred in 16 patients (35.6%) receiving 4 mg and six patients (15.0%) receiving 2 mg (*P* = 0.03). LDA rates were also significantly higher in the 4 mg group, as assessed by CDAI (82.2% vs. 50.0%; *P* = 0.01) and SDAI (80.0% vs. 47.5%; *P* = 0.02). Except for erythrocyte sedimentation rate, all core set outcomes improved significantly more in the 4 mg group (*P* < 0.05). Functional status improved significantly within both groups (*P* < 0.05). Adverse events were generally mild to moderate. Herpes zoster occurred in two patients (4.5%) in the 4 mg group and one patient (2.5%) in the 2 mg group. No cases of tuberculosis, malignancy, venous thromboembolism, or death were observed.

**Conclusions:**

In patients with moderate-to-severe RA and an inadequate response to methotrexate, 4 mg baricitinib demonstrated superior efficacy compared with 2 mg baricitinib, with a comparable safety profile over 24 weeks.

**Trial registration:**

This study was registered with ClinicalTrials.gov (Identifier: NCT05660655, https://clinicaltrials.gov/ct2/show/NCT05660655) on 13 December 2022.

## Introduction

### Background and rationale

Rheumatoid arthritis (RA) is a chronic inflammatory disease that affects joints and systemic health; if inadequately treated, it can lead to joint damage and disability [1, 2]. Methotrexate remains the cornerstone of initial disease-modifying antirheumatic drug (DMARD) therapy; however, a substantial proportion of patients fail to achieve adequate disease control or are unable to tolerate methotrexate despite optimized dosing [3, 4]. In such cases, timely escalation of therapy is essential to prevent irreversible joint damage and long-term disability [5].

Targeted synthetic DMARDs, particularly Janus kinase (JAK) inhibitors, have emerged as effective treatment options for patients with moderate to severe RA [6]. Baricitinib is an oral, selective JAK1/JAK2 inhibitor that disrupts cytokine signaling pathways central to RA pathogenesis. Clinical trials conducted predominantly in high-income countries have demonstrated that baricitinib, either as monotherapy or in combination with methotrexate, significantly improves clinical, functional, and radiographic outcomes in patients with inadequate response to conventional synthetic DMARDs [7–9].

Baricitinib is approved at doses of 2 mg and 4 mg daily, with evidence suggesting dose-dependent efficacy. The 4 mg dose has been associated with higher rates of disease control, whereas the 2 mg dose may offer a favorable safety profile in certain populations. However, real-world data comparing these doses remains limited, particularly in developing countries where disease characteristics, comorbidities, infection risks, and healthcare access may differ from those in high-income settings. Furthermore, concerns about adverse events, such as infections, herpes zoster, and laboratory abnormalities, underscore the need for population-specific safety data [10–15].

In Bangladesh and similar resource-limited settings, access to biologic DMARDs is often restricted due to cost and infrastructure constraints, making oral targeted therapies such as baricitinib an attractive alternative. Despite increasing clinical use, there is a paucity of randomized controlled trial data evaluating the comparative efficacy and safety of different baricitinib doses in this context.

### Objectives

The primary objective of this study was to compare the efficacy of baricitinib 2 mg versus baricitinib 4 mg, each in combination with methotrexate, in achieving low disease activity and remission in patients with moderate-to-severe rheumatoid arthritis who have an inadequate response to methotrexate.

The secondary objectives were to compare the two treatment groups with respect to disease activity assessed by multiple composite indices (DAS28-ESR, CDAI, and SDAI), functional outcomes measured by the Bangla version of the Health Assessment Questionnaire–Disability Index (B-HAQ), changes in individual core set measures and acute-phase reactants, and to evaluate and compare the safety and tolerability profiles of the two dosing regimens over 24 weeks.

## Materials and methods

### Study Design and Setting

This was a 24-week, open-label, randomized, parallel-group, controlled clinical trial conducted at the outpatient Department of Rheumatology at Bangladesh Medical University (BMU; formerly Bangabandhu Sheikh Mujib Medical University [BSMMU]), a tertiary referral center in Bangladesh. The trial was conducted between 1 October 2022 and 30 September 2023 in accordance with the CONSORT 2010 guidelines.

### Participants

Adults aged ≥ 18 years fulfilling the 2010 ACR/EULAR classification criteria for rheumatoid arthritis (RA) [14] with moderate-to-severe disease activity (DAS28-CRP > 3.2) were eligible. All participants had received methotrexate (MTX) at optimized doses (20–25 mg/week for at least 12 weeks), but had demonstrated an inadequate response or intolerance.

**Exclusion criteria** included: active or recent infections (including tuberculosis), hemoglobin < 8 g/dL, white blood cell count < 4,000/mm³, absolute neutrophil count < 1,200/mm³, platelet count < 100,000/mm³, recent live vaccines (< 3 months), estimated glomerular filtration rate < 60 mL/min/1.73 m², alanine aminotransferase > 3× upper limit of normal, history of thromboembolism or diverticulitis, pregnancy, breastfeeding, or inadequate contraception in women of childbearing potential. Only patients with established RA were included in this study; patients with early or newly diagnosed RA were excluded.

**Randomization and Allocation**: Eligible participants were randomized 1:1 to baricitinib 2 mg or 4 mg daily, each in combination with methotrexate 10 mg/week, using a computer-generated random sequence. Allocation was concealed using sealed, opaque, sequentially numbered envelopes prepared by an independent investigator. Study investigators enrolled participants and opened envelopes sequentially to reveal treatment assignment. Due to dosing differences, the study was open-label.

**Interventions Baricitinib 2 mg group**: 2 mg orally once daily + methotrexate 10 mg/week, and **Baricitinib 4 mg group**: 4 mg orally once daily + methotrexate 10 mg/week.

All patients received folic acid supplementation. Stable doses of NSAIDs and adjuvant analgesics were permitted. Oral glucocorticoids were tapered and discontinued whenever possible. The MTX dose was maintained at 10 mg/week throughout the trial to minimize adverse effects. Before enrollment, all patients received methotrexate at escalating doses up to 20 mg/week for at least 12 weeks to ensure adequate optimization. Baricitinib was added only in those with inadequate response to methotrexate. During the trial, the methotrexate dose was maintained at 10 mg/week in combination with baricitinib to reduce the risk of adverse effects, in line with local clinical practice and safety considerations.

**Outcomes: Primary outcome**: Proportion of patients achieving **low disease activity (LDA) by DAS28-CRP** at week 24.**Secondary outcomes**: Disease activity by DAS28-ESR, CDAI, and SDAI, Changes from baseline in individual ACR core set measures and acute-phase reactants, Functional improvement measured using the Bangla version of the Health Assessment Questionnaire–Disability Index (B-HAQ), with a reduction of ≥ 0.22 considered clinically meaningful, Safety and tolerability, including adverse events, serious adverse events, laboratory abnormalities, and infections.

### Sample Size

Based on published remission and LDA rates with baricitinib 2 mg and 4 mg, a minimum of 43 patients per group was required for 80% power at a two-sided 5% significance level. To account for a 10% dropout rate, 47 patients per group (total *n* = 94) were enrolled.

**Compliance with CONSORT Guidelines**: This randomized controlled trial was conducted in accordance with the CONSORT (Consolidated Standards of Reporting Trials) guidelines. **Statistical Methods**: Analyses were performed using SPSS version 25. Continuous variables were expressed as mean ± standard deviation or median, and categorical variables as frequencies and percentages. Normality was assessed using the Shapiro–Wilk test. Between-group comparisons used unpaired t-tests or Mann–Whitney U tests; within-group comparisons used paired t-tests or Wilcoxon signed-rank tests. Categorical outcomes were analyzed using chi-square or Fisher’s exact tests. Two-sided P values ≤ 0.05 were considered statistically significant. Missing primary outcome data were handled using last-observation-carried-forward.

### Ethical Considerations

The study was approved by the Institutional Review Board/Ethics Committee of BSMMU (IRB No. 2022/11646). Written informed consent was obtained from all participants. The trial was registered at ClinicalTrials.gov (NCT05660655).

### Participant Flow and Baseline Characteristics

**Fig. 1:**
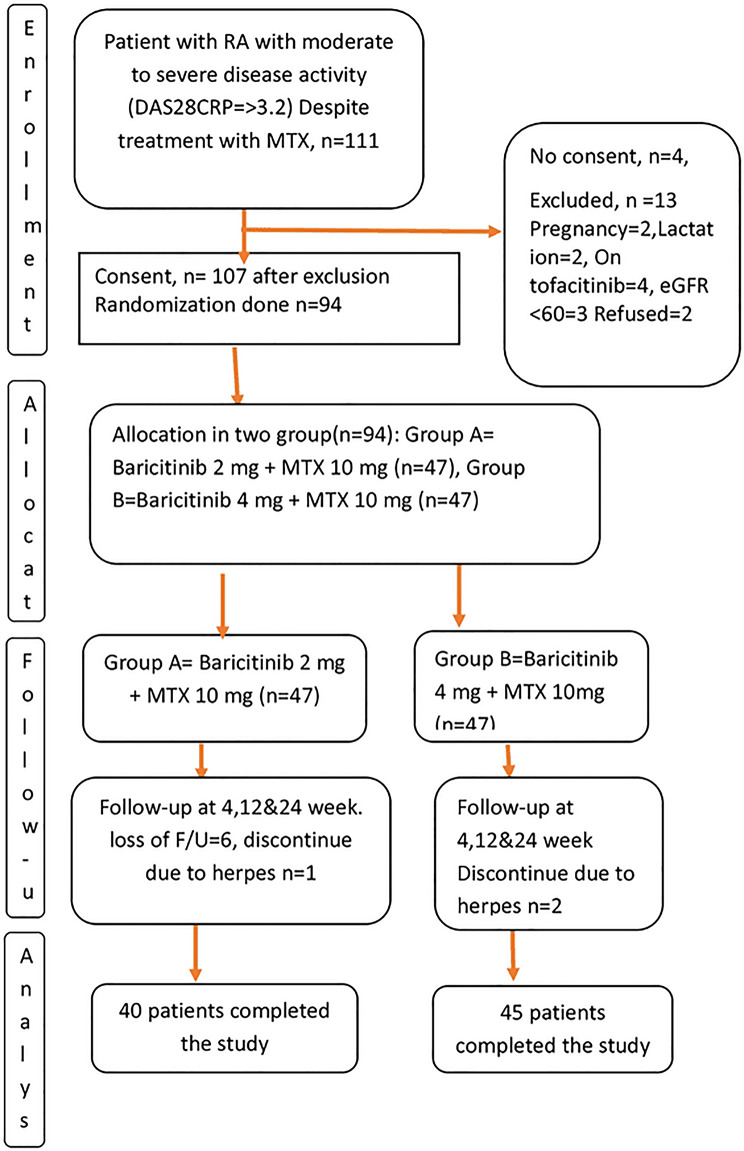
Flow diagram of study population in the randomized controlled trial *N* = 111

Baseline serological status and prior DMARD exposure were recorded. Rheumatoid factor (RF) and anti-citrullinated protein antibody (ACPA) positivity were balanced between groups. All participants had received optimized MTX therapy for ≥ 12 weeks. Baseline prednisone was taken by 2 in the 2 mg group and 3 in the 4 mg BARI group. The flow of participants through the study is illustrated in Fig. 1, in accordance with CONSORT guidelines.

## Results

### Participant Flow

A total of 111 patients with rheumatoid arthritis were assessed for eligibility. Seventeen patients were excluded for not meeting the inclusion criteria or for meeting the exclusion criteria. The remaining 94 patients were randomized 1:1 to receive either baricitinib 2 mg plus methotrexate 10 mg weekly (*n* = 47) or baricitinib 4 mg plus methotrexate 10 mg weekly (*n* = 47).

All randomized participants received at least one dose of the assigned intervention and were included in the **safety population**. During the 24-week follow-up, 9 patients were lost to follow-up: 7 in the 2 mg group and 2 in the 4 mg group (withdrawal of consent, *n* = 4; non-attendance at scheduled visits, *n* = 5). Consequently, **85 patients completed the study** and were included in the **per-protocol efficacy analysis** (2 mg, *n* = 40; 4 mg, *n* = 45). Participant flow is illustrated in Fig. 1.

### Recruitment and Adherence

Participants were recruited consecutively from the Outpatient Department of Rheumatology at Bangabandhu Sheikh Mujib Medical University, Bangladesh, between 1 October 2022 and 30 September 2023. Follow-up was completed for all participants as planned. Oral baricitinib (2 mg or 4 mg daily) was administered in combination with weekly methotrexate 10 mg. Adherence was assessed at each visit via self-report and pill counts and was satisfactory in both groups. Stable doses of NSAIDs and low-dose corticosteroids (≤ 10 mg/day prednisolone equivalent) were permitted and remained comparable between groups.

### Baseline Characteristics

Demographic, clinical, and laboratory characteristics were well balanced between groups (Table 1). Mean age was 41.7 ± 10.4 years in the 2 mg group and 43.3 ± 10.7 years in the 4 mg group. Most participants were female and homemakers. Median disease duration was 6 years (IQR 3–10) in the 2 mg group and 5 years (IQR3–8) in the 4 mg group. Baseline tender and swollen joint counts, patient and physician global assessments, ESR, CRP, composite disease activity indices (DAS28-ESR, DAS28-CRP, CDAI, SDAI), and functional status (B-HAQ) were comparable. Extra-articular manifestations were absent, and smoking prevalence was low (two patients per group).

**Table 1:** Baseline characteristics of study participants (*n* = 94)

Variable	BARI 2 mg + MTX 10 mg (*n* = 47)	BARI 4 mg + MTX 10 mg (*n* = 47)	*P*-value
Gender, n (%)			0.646¹
Male	2 (4.3)	3 (6.4)	-
Female	45 (95.7)	44 (93.6)	-
Smoking, n (%)	2 (4.3)	2 (4.3)	1¹
Occupation, n (%)			0.61¹
Homemaker	39 (83.0)	37 (78.7)	-
Service	6 (12.8)	9 (19.1)	-
Unemployed	2 (4.3)	1 (2.1)	-
Age (years), mean ± SD	41.66 ± 10.37	43.30 ± 10.69	0.452²
BMI (kg/m²), mean ± SD	24.2 ± 4.2	23.7 ± 3.6	0.562²
Disease duration (years), median (IQR)	6.0 (3.0–10.0)	5.0 (3.0–8.0)	0.543³
Tender joints count (0–28), mean ± SD	14.09 ± 5.91	14.06 ± 5.88	0.986²
Swollen joints count (0–28), median (IQR)	2.0 (1.0–3.0)	2.0 (1.0–3.0)	0.369³
PtGA (VAS 0–10 cm), mean ± SD	6.55 ± 1.25	6.38 ± 1.26	0.512²
PhGA (VAS 0–10 cm), mean ± SD	6.19 ± 1.21	5.91 ± 1.18	0.264²
B-HAQ, mean ± SD	1.57 ± 0.49	1.48 ± 0.62	0.421²
DAS28-ESR, mean ± SD	5.87 ± 0.82	5.72 ± 0.85	0.400²
DAS28-CRP, mean ± SD	5.20 ± 0.79	5.16 ± 0.74	0.800²
CDAI, median (IQR)	27.0 (21.0–35.0)	27.0 (22.0–35.0)	0.697³
SDAI, median (IQR)	30.40(23.60–36.11)	29.13(22.77–37.25)	0.636³
ESR (mm/h), median (IQR)	40.0 (25.0–70.0)	40.0 (20.0–62.0)	0.702³
CRP (mg/L), median (IQR)	14.2 (6.7–29.1)	12.5 (5.8–25.0)	0.694³
Hemoglobin (g/dL), mean ± SD	11.2 ± 1.4	11.0 ± 1.5	0.419²
RF positive, n (%)	36 (76.6)	34 (72.3)	0.626⁴
Anti-CCP positive, n (%)	38 (80.9)	37 (78.7)	0.788⁴

1 **=** χ² test or Fisher’s exact test for categorical variables,2 = Independent t-test for normally distributed continuous variables,3 = Mann–Whitney U test for non-normally distributed continuous variables,4 = Fisher’s exact test for small cell counts

### Efficacy at Week 24

#### Between-Group Comparisons

At 24 weeks, patients receiving baricitinib 4 mg plus methotrexate showed significantly greater improvement than those receiving 2 mg across most core set outcomes and composite indices (Table 2). Tender and swollen joint counts, patient and physician global assessments, CRP levels, and B-HAQ scores were lower in the 4 mg group (*P* < 0.05). ESR reductions did not differ significantly (*P* = 0.203). Composite disease activity scores (DAS28-ESR, DAS28-CRP, CDAI, SDAI) were significantly improved in the 4 mg group (*P* < 0.001 for all).

**Table 2:** Comparison of core set outcomes and composite disease activity measures between treatment groups at week 24 (*n* = 85)

Variables	BARI 2 mg + MTX 10 mg group (*n* = 40)	BARI 4 mg + MTX 10 mg group (*n* = 45)	*P*-value
Tender joints (0–28)	4.0 (2.0-6.75)	2.0 (1.0–4.0)	0.004##
Swollen joints (0–28)	0.0 (0.0–1.0)	0.0 (0.0–0.0)	0.026##
PtGA, VAS 0–10 cm	3.50 (2.0–5.0)	2.0 (1.0–3.0)	< 0.001##
PhGA, VAS 0–10 cm	3.0 (2.0–4.0)	2.0 (0.0–2.0)	< 0.001##
ESR (mm/h)	41.0 (27.50–63.50)	34.0 (23.50–46.0)	0.203##
CRP (mg/L)	14.20 (6.70–29.10)	12.51 (5.83-25.0)	0.038##
B-HAQ	0.38 (0.13–0.84)	0.13 (0.0-0.25)	< 0.001##
DAS28-ESR	4.19 ± 0.87	3.51 ± 0.94	< 0.001#
DAS28-CRP	3.43 (2.63–3.97)	2.67 (1.93-3.0)	< 0.001##
CDAI	11.0 (6.0–16.0)	6.0 (2.0–9.0)	< 0.001##
SDAI	11.92 (7.17–17.34)	6.65 (2.40–9.47)	< 0.001##

Data are presented as median (interquartile range) unless otherwise indicated; DAS28-ESR is presented as mean ± standard deviation. BARI = Baricitinib; MTX = Methotrexate; B-HAQ = Bangla version of the Health Assessment Questionnaire Disability Index; PtGA = Patient Global Assessment of disease activity; PhGA = Physician Global Assessment of disease activity; VAS = Visual Analogue Scale; CRP = C-reactive protein; ESR = erythrocyte sedimentation rate; n = number of patients. # Independent Student’s t-test; ## Mann–Whitney U test

**Within-Group Comparisons**: Both treatment groups demonstrated significant improvements from baseline to week 24 in tender and swollen joint counts, PtGA, PhGA, CRP, B-HAQ, and composite indices (*P* < 0.001 for all). ESR did not improve significantly within either group. Mean hemoglobin levels increased modestly in both groups (2 mg: 11.2 → 11.4 g/dL; 4 mg: 11.0 → 11.2 g/dL), consistent with disease control (Table 3).

**Table 3:** Within-group changes in core outcomes and composite disease activity measures from baseline to week 24

Outcome variable	Baseline (*n* = 47)	24th week (*n* = 40)	*p*-value	Baseline (*n* = 47)	24th week (*n* = 45)	*P*
TJC	14.45 ± 5.78	4.68 ± 3.65	< 0.00a	13.73 ± 5.79	2.67 ± 2.62	< 0.001a
SJC	2.33 ± 2.09	0.35 ± 0.62	< 0.00a	1.84 ± 1.61	0.11 ± 0.38	< 0.001a
PtGA	6.55 ± 1.24	3.35 ± 1.69	< 0.00a	6.33 ± 1.26	2.07 ± 1.54	< 0.001a
PhGA	6.23 ± 1.19	2.83 ± 1.43	< 0.00a	5.87 ± 1.16	1.76 ± 1.37	< 0.001a
ESR	47.15 ± 28.29	44.75 ± 26.36	0.39b	43.73 ± 27.97	37.87 ± 21.81	0.260b
CRP	19.91 ± 19.12	11.65 ± 23.94	< 0.00b	18.29 ± 18.71	4.75 ± 5.77	< 0.001b
B-HAQ	1.60 ± 0.49	0.45 ± 0.37	< 0.00a	1.45 ± 0.61	0.23 ± 0.34	< 0.001a
Composite measures						
DAS28-ESR	5.89 ± 0.84	4.18 ± 0.87	< 0.00a	5.67 ± 0.84	3.51 ± 0.94	< 0.001a
DAS28-CRP	5.21 ± 0.77	3.28 ± 1.02	< 0.00a	5.12 ± 0.72	2.62 ± 0.92	< 0.001a
CDAI	29.33 ± 7.93	11.18 ± 6.67	< 0.00a	27.71 ± 7.92	6.67 ± 5.55	< 0.001a
SDAI	31.20 ± 8.35	12.34 ± 7.53	< 0.00a	29.49 ± 8.35	7.13 ± 5.88	< 0.001a

Baseline values include all randomized participants; week-24 values include only participants with complete follow-up. TJC: Tender joint count, SJC: Swollen joint count, PtGA: Patient Global Assessment (0–10 scale), PhGA: Physician Global Assessment (0–10 scale), ESR: Erythrocyte sedimentation rate (mm/hr), CRP: C-reactive protein (mg/L), B-HAQ: Bangla version of the Health Assessment Questionnaire Disability Index, Composite measures: DAS28-ESR, DAS28-CRP, CDAI, SDAI. ᵃ: Paired t-test for normally distributed data. ᵇ: Wilcoxon signed-rank test for non-normally distributed data. P-values < 0.05 were considered statistically significant

#### Mean Change from Baseline

The mean reduction in DAS28-CRP was greater in the 4 mg group than in the 2 mg group (2.5 [95% CI 2.2–2.8] vs. 1.9 [95% CI 1.6–2.3]; *P* = 0.01). Mean improvements in PtGA and PhGA were also significantly larger in the 4 mg group (Table 4).

**Table 4:** Between-group mean change from baseline at Week 24 (per-protocol population, *n* = 85)

Variables	2 mg + MTX (*n* = 40) Mean Δ (95% CI)	4 mg + MTX (*n* = 45) Mean Δ (95% CI)	*P* value
Core set outcomes			
Tender joint count (0–28)	9.7(8.2–11.3)	11.1 (9.2–12.9)	0.50‡
Swollen joint count (0–28)	1.9 (1.4–2.6)	1.7 (1.2–2.2)	0.77‡
PtGA	3.2 (2.6–3.8)	4.2 (3.7–4.8)	0.01†
PhGA	3.4 (2.9–3.8)	4.1 (3.6–4.6)	0.03‡
ESR	2.4 (− 8.3–13.1)	5.8 (− 3.6–15.3)	0.62†
CRP	8.2 (0.7–15.8)	13.5 (8.1–19.0)	0.18‡
B-HAQ	1.1 (0.9–1.3)	1.2 (1.0–1.4)	0.62‡
Composite measures			
DAS28-ESR	1.7 (1.4–2.0)	2.2 (1.8–2.5)	0.06‡
DAS28-CRP	1.9 (1.6–2.3)	2.5 (2.2–2.8)	0.01†
CDAI	18.2(15.7–20.6)	21.0 (18.3–23.8)	0.12†
SDAI	18.9(16.1–21.6)	22.4 (19.4–25.3)	0.08†

Δ = mean change from baseline to week 24; 95% CI = 95% confidence interval; † Independent Student’s t-test (parametric); ‡ Mann–Whitney U test (non-parametric). PtGA = Patient Global Assessment; PhGA = Physician Global Assessment; B-HAQ = Bangla Health Assessment Questionnaire–Disability Index; CRP = C-reactive protein; ESR = erythrocyte sedimentation rate; DAS28 = Disease Activity Score in 28 joints; CDAI = Clinical Disease Activity Index; SDAI = Simplified Disease Activity Index; MTX = Methotrexate

#### Low Disease Activity

**Fig. 2:**
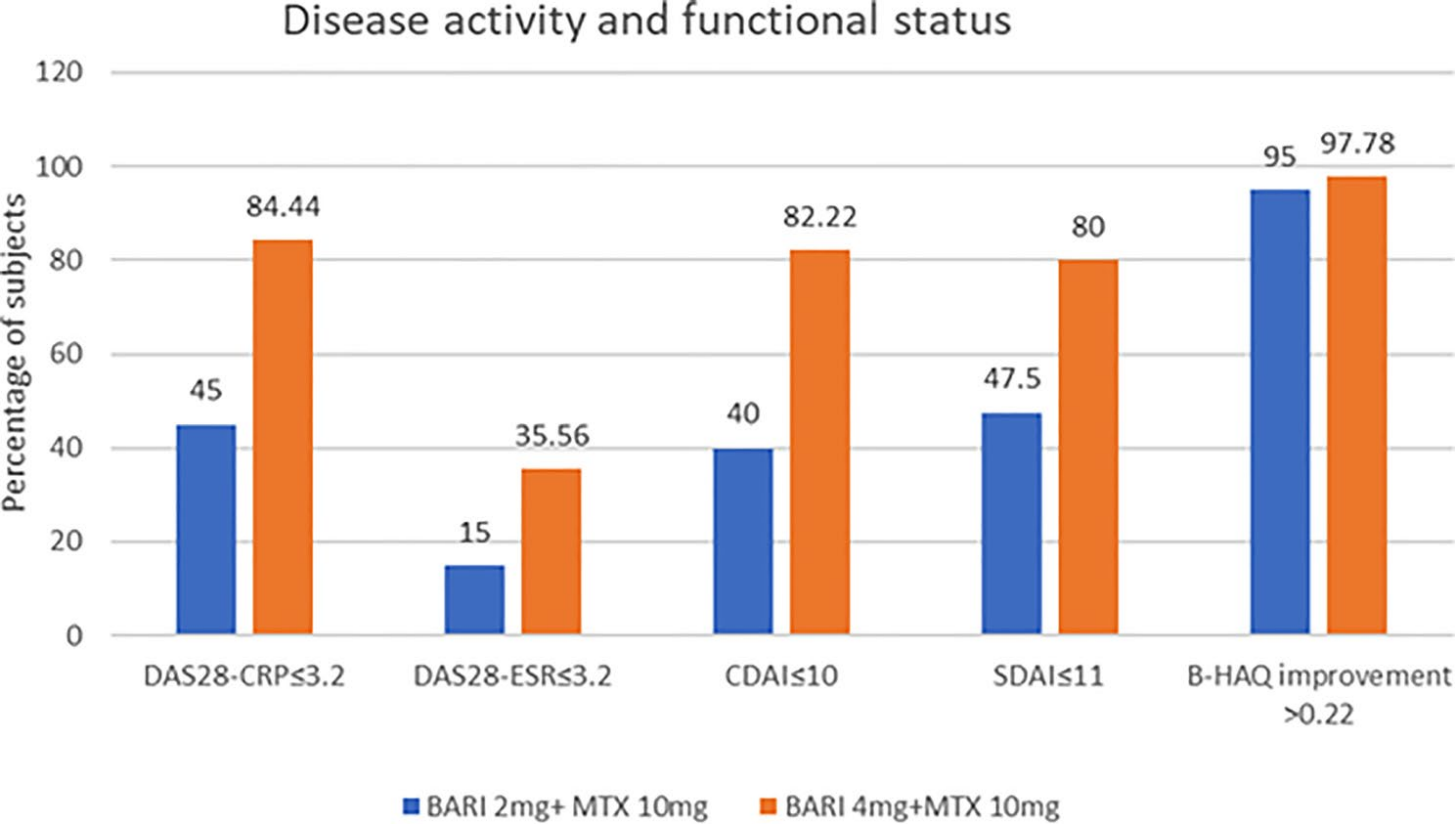
Bar diagram showing disease activity (per-protocol analysis) and functional status between the groups at the 24^th^

Using DAS28-CRP criteria, low disease activity (LDA) at week 24 was achieved by **38/45 patients (84.4%)** in the 4 mg group versus **18/40 patients (45.0%)** in the 2 mg group (*P* = 0.0001). Differences were also significant when assessed using DAS28-ESR, CDAI, and SDAI. Both groups achieved clinically meaningful improvements in B-HAQ, with no statistically significant difference between them. Disease activity and functional outcomes are illustrated in Fig. 2.

**Safety and Adverse Events**: A total of 27 adverse events occurred in the 2 mg group and 38 in the 4 mg group. At least one adverse event was reported by 18 patients (45.0%) in the 2 mg group and 26 patients (57.8%) in the 4 mg group (*P* = 0.239). Serious adverse events were infrequent (2 mg: 4/40, 10.0%; 4 mg: 3/45, 6.7%), including herpes zoster infections. Withdrawals due to adverse events occurred in 1 patient in the 2 mg group and 2 in the 4 mg group. The most common adverse events were skin eruptions or itching, more frequent in the 4 mg group, but not statistically significant. Gastrointestinal events, infections, renal and hematologic abnormalities were comparable between groups. Elevated ALT was observed only in the 4 mg group. No cases of tuberculosis, venous thromboembolism, malignancy, or death occurred. Overall, safety profiles were comparable between groups (Table 5).

**Table 5:** Comparison of adverse events between the 2 mg and 4 mg treatment groups

Adverse Event Category	BARI 2 mg + MTX (*n* = 40), n (%)	BARI 4 mg + MTX (*n* = 45), n (%)	*P*-value*
Patients with ≥ 1 adverse event	18 (45.0)	26 (57.8)	0.239
Serious adverse events (SAE)	4 (10.0)	3 (6.7)	0.577
Withdrawal due to adverse event	1 (2.5)	2 (4.4)	0.641
Herpes zoster	1 (2.5)	2 (4.4)	0.641
**Infections**			
Fever	4 (10.0)	5 (11.1)	1.000
Nasopharyngitis	3 (7.5)	4 (8.9)	1.000
Urinary tract infection	2 (5.0)	1 (2.2)	0.488
**Skin and Subcutaneous Tissue Disorders**			
Skin eruptions/itching	5 (12.5)	11 (24.4)	0.084
**Gastrointestinal Disorders**			
Nausea/vomiting	2 (5.0)	3 (6.7)	1.000
Abdominal pain	2 (5.0)	1 (2.2)	0.488
**Nervous System Disorders**			
Headache	3 (7.5)	5 (11.1)	0.47
Hepatic Disorders			
Elevated serum ALT	0 (0.0)	4 (8.9)	0.058
**Renal Disorders**			
Elevated serum creatinine	3 (7.5)	1 (2.2)	0.251
Acute kidney injury	0 (0.0)	1 (2.2)	1.000
**Hematologic Disorders**			
Neutropenia (< 1000/µL)	1 (2.5)	0 (0.0)	1.00
**Cardiovascular Disorders**			
Hypertension	1 (2.5)	0 (0.0)	1.00

* P-values are exploratory and not adjusted for multiple comparisons

%, percentage of patients, Abbreviations: ALT=alanine aminotransferase; BARI= Baricitinib, MTX= methotrexate

#### Low Disease Activity

At week 24, a significantly higher proportion of patients in the baricitinib 4 mg group achieved low disease activity (LDA) than in the 2 mg group. According to the DAS28-CRP criteria, 84.4% of patients in the 4 mg group reached LDA, compared with 45.0% in the 2 mg group. Both the absolute risk difference and relative risk indicate a clinically meaningful benefit of the higher dose (Table 6).

**Table 6:** Proportion of patients achieving low disease activity according to DAS28-CRP at Week 24

Outcome	BARI 2 mg + MTX, *n*/*N* (%)	BARI 4 mg + MTX, *n*/*N* (%)	Risk Difference (95% CI)	Relative Risk (95% CI)	*P*-value
LDA (DAS28-CRP)	18/40 (45.0%)	38/45 (84.4%)	39.4% (20.5–58.3)	1.87 (1.32–2.64)	< 0.001

**DAS28-CRP**: Proportion of patients achieving low disease activity according to the Disease Activity Score in 28 joints using C-reactive protein. **BARI 2 mg + MTX**: Baricitinib 2 mg in combination with methotrexate. **BARI 4 mg + MTX**: Baricitinib 4 mg in combination with methotrexate. Risk Difference and Relative Risk are presented with 95% confidence intervals (CI). P-values < 0.05 were considered statistically significant.

#### Ancillary Analyses

No prespecified subgroup or exploratory analyses were conducted.

## Discussion

In this study, patients with RA who were not responding to methotrexate demonstrated significant improvement in the core set of outcomes in the baricitinib 4 mg group compared to the 2 mg group, except for ESR. The primary endpoint of achieving low disease activity (LDA) based on DAS28-CRP was significantly higher in the 4 mg group. A higher proportion of patients reached remission or LDA in the 4 mg group, consistent with the findings of Keystone et al. [16]. Both treatment groups showed significant within-group improvement in tender and swollen joint counts, patient and physician global assessments, and acute-phase reactants (except ESR), accompanied by improvements in composite activity measures at 24 weeks. These findings align with those reported by Dougados et al. [17]. The lack of significant ESR change from baseline to week 24, both within and between groups, also mirrors the observations of Keystone et al. [16]. A potential explanation may be the predominance of female participants, as ESR varies by sex and is generally higher in females.

Regarding achieving DAS28-CRP remission or LDA, 45% of patients in the 2 mg group achieved this outcome, consistent with the 46% reported in the RA-BUILD trial [17]. In another study, 4 mg of baricitinib achieved LDA in 81.6% of patients [18], which is comparable to our findings. Bae et al. [19] further demonstrated that baricitinib 4 mg plus methotrexate is more effective than 2 mg plus methotrexate in moderate to severe RA, supporting the results of the present study.

Regarding safety, no major differences were observed in the incidence of serious adverse events (SAEs) or serious infections between the groups. Herpes zoster infection occurred in 4.4% of patients in the 4 mg group and 2.5% in the 2 mg group, comparable to the 4% incidence reported by Genovese et al. [20] for baricitinib 4 mg. Skin eruptions or itching were the most common adverse events in both groups, occurring in 24.44% of the 4 mg group and 12.5% of the 2 mg group.

One patient in the 2 mg group developed new-onset hypertension. Headache and elevated ALT were more frequent in the 4 mg group, whereas urinary tract infection, elevated serum creatinine, and abdominal pain were more common in the 2 mg group. One patient in the 2 mg group had a neutrophil count < 1000/L (Grade 3), prompting treatment suspension. Neutrophil counts < 1000/L are relatively uncommon (< 1%) [21]; in this study, they occurred in 2.5%, possibly due to the small sample size. One patient in the 4 mg group experienced acute kidney injury requiring hospitalization; treatment was temporarily discontinued and later resumed after renal function normalized. Both groups exhibited modest increases in lymphocytes and triglycerides, with reductions in neutrophils. In the 4 mg group, increases in LDL cholesterol and serum creatinine were also noted. These findings are consistent with Fleischmann et al. [22], although their study used a higher methotrexate dosage (20 mg/week). Neither hemoglobin nor platelet counts significantly changed in either treatment group.

### Limitations

This study had an open-label design, a modest sample size, and a short follow-up, which may have limited the ability to detect rare adverse events and assess long-term efficacy. Nevertheless, this is the first study to directly compare baricitinib 2 mg plus methotrexate 10 mg with 4 mg plus methotrexate 10 mg in this patient population.

### Conclusion

Baricitinib 4 mg plus methotrexate 10 mg demonstrated greater efficacy than 2 mg in achieving low disease activity and improving core outcomes in patients with rheumatoid arthritis inadequately responding to methotrexate, with a comparable safety profile. Both doses were generally well tolerated, and no unexpected safety signals were observed during the study period. Longer-term studies are warranted to assess sustained efficacy and long-term safety.

## Data Availability

The data supporting the conclusions of this study are available from the corresponding author upon reasonable request.

## Abbreviations

ACPA: Anti-citrullinated protein antibodies
ACR: American College of Rheumatology
BHAQ: Bengali Health Assessment Questionnaire
BMU: Bangladesh Medical University
CDAI: Clinical Disease Activity Index
CRP: C-reactive protein
DAS 28-CRP: Disease Activity Score-28 for Rheumatoid Arthritis with CRP
ESR: Erythrocyte Sedimentation Rate
EULAR: The European Alliance of Associations for Rheumatology
IGRA: Interferon-Gamma Release Assay
LDA: Low Disease Activity
Pt GA: Patient Global Assessment
SDAI: Simplified Disease Activity Index
SJC: Swollen Joint Count
TJC: Tender Joint Count
